# Liposomal Amphotericin B Treatment and the Leishmaniases

**DOI:** 10.4269/ajtmh.19-0568

**Published:** 2019-09-03

**Authors:** Jonathan Berman

**Affiliations:** Fast-Track Drugs and Biologics, North Potomac, Maryland

Visceral leishmaniasis (VL), characterized by fever, hepatosplenomegaly, and pancytopenia, is considered fatal if untreated. The largest number of cases worldwide has classically been in the northeast of the Indian subcontinent (Northeast India, Bangladesh, and Nepal) and is due to anthropomorphic *Leishmania* (*L.*) *donovani.* Since ∼2,000, VL chemotherapy in the Indian subcontinent has been transformed from multiple parenteral administrations of pentavalent antimony, pentamidine, or amphotericin B deoxycholate to oral miltefosine and then to liposomal amphotericin B (L-AmB). Because L-AmB is less toxic than amphotericin B deoxycholate, instead of administering the standard 15 mg AmB/kg total dose as 1 mg AmB deoxycholate/kg every other day over 30 days (Table 1, study 1), the total dose can be administered via much larger individual doses over much shorter periods of time. Ten years ago, in a signal publication, Sundar et al.^1^ showed that 10 mg L-AmB/kg administered only once cured 96% of 304 patients with 6-month follow-up (Table 1, study 2). In this issue of the journal, the effect of this regimen, one injection of 10 mg/kg L-AmB, is reported in a larger study of 1,143 patients with longer (12-month) follow-up.^2^ At 1, 6, and 12 months, the cure rates were 100%, 97%, and 94%, respectively, by per protocol analysis (Table 1, study 3). Male gender, weight less than 30 kg, and spleen size greater than 4 cm were independently associated with failure.

**Table 1:** Effect of liposomal amphotericin B (L-AmB) on VL, CL, and ML: representative studies

Study No.	Disease (*Leishmania* species)	Endemic region	L-AmB regimen	Total dose	No. of patients	% Cure (month assessed)	Ref.
1	VL (*L. donovani*)	Indian subcontinent	AmB (deoxycholate) 1 mg/kg qod × 15 injs	15 mg/kg	108	96.3 (6 months)	1
2	VL (*L. donovani*)	Indian subcontinent	10 mg/kg × 1 inj	10 mg/kg	304	95.7 (6 months)	1
3	VL (*L. donovani*)	Indian subcontinent	10 mg/kg × 1 inj	10 mg/kg	1,143	94 (12 months)	2
4	VL (*L. donovani*)	East Africa	10 mg/kg × 1 inj	10 mg/kg	40	58 (6 months)	4
			3 mg/kg × 7 injs over 21 days	21 mg/kg	54	85 (6 months)	4
5	VL (*L. infantum*)	Europe	1–1.4 mg/kg/day × 21 days	21–29 mg/kg	10	100 (12 months)	5
			3 mg/kg/day × 10 days	30 mg/kg	10	100 (12 months)	5
6	VL (*L. chagasi*)	Brazil	3 mg/kg/day × 7 days	21 mg/kg	109	87 (6 months)	7
7	VL (*L. chagasi*)	Brazil	2 mg/kg/day × 7 injs over 10 days	14 mg/kg	13	62 (6 months)	8
8	CL (*L. tropica*)	Old World	3 mg/kg on days 1–5 and 10	18 mg/kg	13	84 (11 months)	12
9	CL (*L. braziliensis*)	New World	3 mg/kg on days 1–5 and 10	18 mg/kg	34	97 (2 years)	13
10	ML	New World	Not available	30–35 mg/kg	16	88 (1 year)	15
11	PKDL	Indian subcontinent	(2 courses of) 5 mg/kg qod × 4 injs	40 mg/kg	4	100	17
12	PKDL	Indian subcontinent	3 mg/kg × 5 injs over 15 days	15 mg/kg	273	78 (12 months)	18

CL = cutaneous leishmaniasis; injs = injections; ML = mucosal leishmaniasis; qod = every other day; PKDL = post–kala-azar dermal leishmaniasis; VL = visceral leishmaniasis.

The spectacular effectiveness of merely one administration of L-AmB for Indian subcontinent VL suggests that the multitude of other leishmaniasis treatment needs might be satisfied by one dose of L-AmB. These needs include elimination of VL from the Indian subcontinent, treatment of VL elsewhere, treatment of cutaneous leishmaniasis (CL) and mucosal leishmaniasis (ML), and treatment of post–kala-azar dermal leishmaniasis.

For elimination of VL from the Indian subcontinent, a key requirement for elimination campaigns is high efficacy after a single (generally yearly) mass drug administration to large populations of whom only some members are infected. The fact that L-AmB is administered intravenously, unfortunately, precludes it from mass drug administration to largely uninfected persons. Other problems with this campaign are reviewed by Singh et al.^3^

Considering non-Indian subcontinent VL, East African VL is, like Indian subcontinent VL, caused by zoonotic *L. donovani*, so it might be thought that East African VL would be equally susceptible to L-AmB. However, 10 mg/kg cured only 58% of East African VL, and the cure rate even with seven injections of 3 mg/kg was 85%—“far less effective than anticipated” (Table 1, study 4).^4^ In the Mediterranean region and in Brazil, VL is due to zoonotic *L. infantum chagasi*. Twenty Europeans who received L-AmB at 1–1.4 mg/kg/day for 21 days or 3 mg/kg/day for 10 days were cured (Table 1, study 5).^5^ The Food and Drug Administration’s (FDA) L-AmB label recommendation for 3 mg/kg on days 1–5, 14, and 21 for a total dose of 21 mg/kg^6^ was probably derived from these data. In Brazil, 3 mg/kg/day for 10 days cured 87% of cases (Table 1, study 6).^7^ That regimen cannot be much reduced because 2 mg/kg on days 1–6 and 10 cured only 62% (Table 1, study 7).^8^

Cutaneous leishmaniasis presents as a papule or nodule that may undergo central ulceration. Most lesions self-cure in a few months for some species (Old World *L. major*,^9^ New World *L. mexicana*^10^) to probably a year or more for other species (Old World *L. tropica*, New World *L. braziliensis*^11^). Three mg/kg given six times over 10 days was effective for Old World *L. tropica* (Table 1, study 8)^12^ and for New World *L. braziliensis* (Table 1, study 9).^13^ New World CL due to *L. braziliensis* and related organisms may uncommonly spread to the mucous membranes of the nose, mouth, larynx, and pharynx, causing ML. Mucosal leishmaniasis does not self-cure,^14^ and with time is clinically devastating as these cartilaginous structures erode. Overall, 87% of patients were cured with mean total doses of 31 mg/kg of L-AmB (Table 1, study 10).^15^ It should be noted that a mean total dose of 20 mg/kg gave low cure rates for all forms of CL and ML seen in France: 58% for Old World CL, 27% for New World CL, and 57% for a few ML cases.^16^

Post–kala-azar dermal leishmaniasis (PKDL), as the name implies, is infection of the skin with *Leishmania* that occurs posttreatment of kala-azar (the colloquial name for VL). In addition to patient benefit, treatment of PKDL removes a parasite reservoir that would otherwise impede elimination efforts. Four cases of PKDL were successfully treated with two 20-mg/kg cycles of L-AmB (Table 1, study 11).^17^ In a larger study of 280 patients treated with a lower regimen of 15 mg/kg (3 mg/kg administered five times over 15 days), the cure rate was 78% (Table 1, study 12).^18^

In sum, treatment of a disease other than Indian subcontinent VL has required total doses and durations of treatment higher than the 10 mg L-AmB/kg for 1 day that offers excellent efficacy for Indian subcontinent VL. For VL, 21 mg/kg of L-AmB cured 85% of East African disease, 30 mg/kg cured 87% of Brazilian disease, and the FDA recommends 21 mg/kg; for these regimens, dosing is over 7–21 days. These regimens of L-AmB may well be used for their respective diseases, but none are strikingly convenient or effective compared with regimens of other drugs. For CL/ML, 18–30 mg/kg may be successful. Because CL is a self-limiting disease, such large intravenous doses over relatively long periods of time are likely to be used only for unusual cases. ML is very difficult to treat—28 days of oral miltefosine achieved a cure rate of about 70%^19^—and the L-AmB cure rates summarized previously are attractive. I expect that L-AmB will be increasingly used for New World ML. If the efficacy of 15 mg L-AmB over 15 days for PKDL is maintained in additional studies, that regimen is easily competitive with the present standard, which is oral miltefosine daily for 12 weeks.^20^ Overall, there are certainly uses for L-AmB for the diverse presentations of leishmaniasis other than Indian subcontinent VL, but unfortunately the effect of L-AmB on them is not as impressive as for Indian subcontinent disease. It is paradoxical that Indian subcontinent VL, the most prevalent form of the most dangerous type of leishmaniasis, is most sensitive to L-AmB, whereas less prevalent forms of VL and less severe types of leishmaniasis are less sensitive to this agent. Additional studies will help us optimize treatments for each syndrome and locality.
